# Atrial fibrillation incidence and outcomes in two cohorts of octogenarians: LiLACS NZ

**DOI:** 10.1186/s12877-023-03902-5

**Published:** 2023-03-30

**Authors:** Ruth Teh, Ngaire Kerse, Avinesh Pillai, Thomas Lumley, Anna Rolleston, Tin Aung Kyaw, Martin Connolly, Joanna Broad, Elaine Monteiro, Valerie Wright-St Clair, Robert N. Doughty

**Affiliations:** 1 Department of General Practice and Primary Health Care, Faculty of Medical and Health Sciences, University of Auckland, PO Box 92019, Auckland, New Zealand; 2 Department of Statistics, Faculty of Science, University of Auckland, Auckland, New Zealand; 3 Manawa Ora, The Centre for Health, Tauranga, New Zealand; 4 Department of Geriatric Medicine, Faculty of Medical and Health Sciences, University of Auckland, Auckland, New Zealand; 5 Centre for Active Ageing, Auckland University of Technology New Zealand, Auckland, New Zealand; 6 Department of Medicine, University of Auckland and Greenlane Cardiovascular Service, Auckland District Health Board, Auckland, New Zealand

**Keywords:** Atrial Fibrillation, Octogenarians, Indigenous Peoples

## Abstract

**Background:**

Atrial fibrillation (AF), the most common cardiac arrhythmia in the general population, has significant healthcare burden. Little is known about AF in octogenarians.

**Objective:**

To describe the prevalence and incidence rate of AF in New Zealand (NZ) octogenarians and the risk of stroke and mortality at 5-year follow-up.

**Design:**

Longitudinal Cohort Study.

**Setting:**

Bay of Plenty and Lakes health regions of New Zealand.

**Subjects:**

Eight-hundred-seventy-seven (379 indigenous Māori, 498 non-Māori) were included in the analysis.

**Methods:**

AF, stroke/TIA events and relevant co-variates were established annually using self-report and hospital records (and ECG for AF). Cox proportional-hazards regression models were used to determine the time dependent AF risk of stroke/TIA.

**Results:**

AF was present in 21% at baseline (Māori 26%, non-Māori 18%), the prevalence doubled over 5-years (Māori 50%, non-Māori 33%). 5-year AF incidence was 82.6 /1000-person years and at all times AF incidence for Māori was twice that of non-Māori. Five-year stroke/TIA prevalence was 23% (22% in Māori and 24% non- Māori), higher in those with AF than without. AF was not independently associated with 5-year new stroke/TIA; baseline systolic blood pressure was. Mortality was higher for Māori, men, those with AF and CHF and statin use was protective.

In summary, AF is more prevalent in indigenous octogenarians and should have an increased focus in health care management. Further research could examine treatment in more detail to facilitate ethnic specific impact and risks and benefits of treating AF in octogenarians.

**Supplementary Information:**

The online version contains supplementary material available at 10.1186/s12877-023-03902-5.

## Background

Atrial fibrillation (AF) is the most common cardiac arrhythmia occurring in 1–2% of the general population; in those aged 80 + years, up to 18% may have AF [1–3]. AF is responsible for substantial morbidity and mortality in older people, being independently associated with an increased long-term risk of stroke, heart failure, cognitive dysfunction and all-cause mortality, driving a significant public health impact related to increased hospitalisations[3–5]. AF has been attributed to hypertension, heart failure, coronary artery disease, valvar heart disease, obesity, chronic kidney disease (CKD) and diabetes [6–8]. More recently, newer risk factors are emerging that have implications for clinical management, in particular, sleep apnoea and a genetic predisposition [6, 7, 9]. Age is a risk factor for AF, and older age is a consideration in the management and risk for mortality associated with AF [10]. With the ageing demographic, where those over age 80 years are the fastest growing population group, [11]. AF will become an increasingly important clinical issue.

Accurate identification for population based estimates is difficult as up to 13% of AF cases are likely to be undiagnosed [12]. Atrial fibrillation is detected and diagnosed by pulse palpation, cardiac auscultation and ECG recording. However, one of the challenges for AF identification and treatment is its possible intermittent initial nature and asymptomatic presentation [4, 7, 9] that can lead to under-recognition [13]. AF prevalence differs in population subgroups and Māori (New Zealand indigenous people) and Pacific populations have a higher prevalence of AF [14]. Robust research is needed to provide accurate population estimates.

Stroke can be prevented with anticoagulation if AF is accurately identified. Prior to the latest information about risk of aspirin, around 2013 [15], aspirin alone was often considered the treatment of choice for people with atrial fibrillation at risk of bleeding from oral anticoagulants [16]. Dabigatran and rivaroxaban were added to the New Zealand publicly funded formulary in 2011 and 2018 respectively [17, 18].

Recommendations for managing AF in those with stroke are established [19]. Existing stroke is a strong predictor of recurrent stroke at all ages. Stroke can also trigger AF [20] suggesting a cyclical mechanism of increasing risk. The increase in stroke risk associated with AF in younger populations is well described [20]. Cardiovascular mechanisms may differ for people in advanced age, and there is debate about efficacy of management for CVD [21] and CVD risk in those successfully surviving into their 80 s [22–24]. For Māori, indigenous to New Zealand, equity in outcomes is a Government priority. Do the risks associated with AF persist into advanced age, and how do risk differ between ethnic groups? Understanding the importance of AF in the majority of older people without prior stroke would aid refinement of recommendations for screening for AF.

There are no population-based community data on AF in indigenous people overseas or in New Zealand [25] and reports in advanced age are rare. The aim of this study is to describe the prevalence and incidence rate of AF in Māori and non-Māori octogenarians living in New Zealand, along with associated risk of stroke and mortality at 5-year follow-up.

## Methods

### Design and setting

The Life and Living to Advanced Age cohort study in New Zealand (LiLACS NZ) is a longitudinal study of the oldest old in New Zealand [26]. The study was initiated in 2010 and used a population based sampling frame to recruit 937 octogenarians living in one large region including rural and urban areas. At baseline, the sample consisted of 421 Māori aged between 80 and 90 years (born in 1920–1930) and 516 non-Māori aged 85 years (born in 1925). Māori are indigenous people of Aotearoa New Zealand. The overall response rate (defined as the number of people in the eligibility pool who agreed to participate in the study divided by all eligible people) for the study was 57% [26]. The recruitment procedures and response rate have been reported. The study sample was similar to the underlying population on age and gender for Māori and for non-Māori, women were slightly under represented [27]. Smoking rates reported in these cohorts are similar to the national data from same time period [28, 29]. In brief, participants were identified from the electoral roll, health care databases and extensive iwi (tribal), family and personal networks and were recruited by personal invitation from the general practitioner, iwi or community contact. Recruitment occurred between March 2010 and April 2011 within the defined health region boundaries in two health districts in the North Island, New Zealand. This paper reports on 877 participants (379 Māori and 498 non-Māori) who gave consent to access to their hospital records.

## Measures

### Interview and assessment data

Trained interviewers used standardised techniques to conduct structured face-to-face interviews and trained nurses conducted physical assessments. The socio-demographic data (sex, age, education), lifestyle behaviour (smoking and alcohol consumption) and medical history were obtained by self-report through interview [30]. NZ Deprivation index, derived from census based small area characteristics, was used as an indication of socio-economic status. All medications, as taken, were recorded by the interviewers by viewing bottles and packets and were coded using the WHO-ATC coding system. Functional status was determined using the Nottingham Extended Activities of Daily Living (NEADL) [31], cognitive function with the Modified Mini Mental State Examination (3MS) [32], quality of life with the SF-12 physical and mental health subscales [33], and depressive symptom with the Geriatric Depression Scale (GDS-15) [34]. Participants were interviewed at yearly intervals between 2010 and 2016, i.e. 5 years (60 months) follow-up.

Physical assessments completed yearly by research nurses included blood pressure: the average of three lying measurements; Weight, using Tanita scales and Height measured three times using a stadiometer. Blood was drawn for fasting serum glucose. ECGs were completed yearly (see below).

### Medical and hospital records

A search of medical records from the general practitioner (GP) was undertaken to ascertain a list of selected diagnosed conditions. This one-page form was completed by either the practice nurse or a research nurse at the general practice searching the electronic record; this occurred at baseline. Diagnoses were either ascertained from the GP record, from a hospital discharge letter (ICD-9 and ICD-10 codes), or from reading through the medical records at baseline [35].

Administrative hospitalisation discharge diagnosis records from all public and private hospitals were obtained through the New Zealand Health Information Service (NZHIS) within the New Zealand Ministry of Health by matching the participant unique health identifying number, National Health Index (NHI). The cause of hospital admission was obtained through standardised ICD9 and ICD10 coding applied by all New Zealand hospitals, whether participants were discharged alive or died in hospital. Hospitalisation data were used to contribute to baseline status (as below) and to ascertain outcomes over 60 months.

Conditions considered as comorbidities were ascertained from interview and assessment data, and medical and hospital records. CVD events, including coronary artery disease (CAD), congestive heart failure (CHF), peripheral vascular disease (PVD); type 2 diabetes; chronic lung disease or asthma; osteoporosis; rheumatoid arthritis; osteoarthritis; dementia; depression; thyroid disease; renal impairment; cancer (any); Parkinson’s disease; eye diseases; and hypertension were ascertained by cross-checking a combination of sources, i.e. self-report, GP medical records, NZHIS and physical assessment including blood sampling [35].

AF was ascertained each year of the study. An electrocardiograph (ECG) was taken using the Welch Allyn CP200 12 lead ECG monitor by the trained research nurse and read independently by a cardiologist (RND). Atrial fibrillation was ascertained as present from the ECG at the time of LiLACS NZ interviews, in the GP record, or from the hospitalisation records. At baseline: ever having AF was if hospital or GP records indicated AF prior to date of enrolment. At each interview, if the ECG documented AF or AF was recorded in hospitalisation records, participant status was updated to ‘ever AF’.

Outcomes of stroke/TIA were established from the hospitalisation record ICD-9 (430, 431, 432.x, 433.xx, 434.xx, 436, 437.xx, 438.xx) and ICD-10 (I60, I61, I62.x, I63.x, I65.x, I66.x, I67.x, I69.x) primary coded diagnoses from the time of enrolment to 5 years follow-up. All-cause mortality was ascertained from national administrative data held by the Ministry of Health using NHI matching.

### Statistical analysis

Descriptive statistics were presented for all variables, frequency (percent) for categorical data and mean (standard deviation, SD) for data with parametric distribution and median (interquartile range, IQR) for non-parametric distribution. Period prevalence, i.e. accruing cases of AF whether the participant withdrew from the study or died, was calculated over 5 years.

The association of variables, outcomes and ethnicity with AF at baseline were examined using the Chi-square test.

Time dependent Cox proportional-hazards regression models were used to determine the risk of stroke/TIA and mortality over the follow-up period. AF is a time-dependent variable since it can change value over the course of our observation period of 60 months. The value of AF could be held fixed at a certain point in time, say baseline or 60 months, but modelling the changing values of AF ensures use of all data available giving a more detailed and powerful analysis. The time to AF was calculated at each year and was modelled as a time to event variable. Since we are interested in the risk for primary stroke as an outcome related to presence of AF, participants with stroke prior to baseline assessment were not included in the analysis.

Multivariate analyses were performed, and the adjusted HRs are reported. The selection of variables included in the Cox regression models was informed by the literature of known factors associated to stroke and AF. None with missing data were included in the analysis. All tests were two sided and were conducted using an alpha value of 0.05. Data were analysed using SAS V.9.4 (SAS Institute) and SPSS 25.0 for Windows.

We seek to identify inequities, and present ethnic specific analyses to enable ethnic specific responses.

## Results

**Fig. 1:**
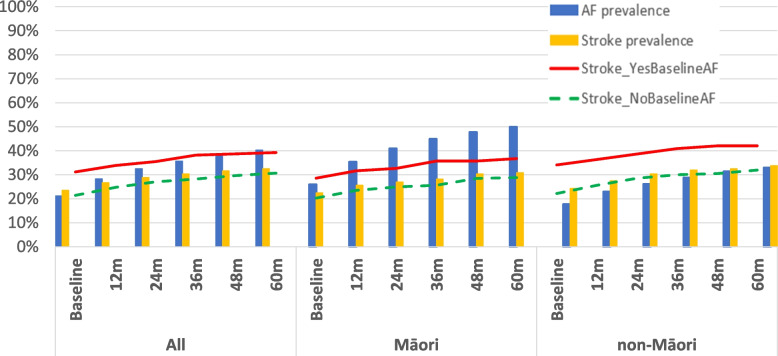
Period prevalence for atrial fibrillation and stroke by ethnic group over 5-year follow-up for octogenarians in New Zealand. AF, Atrial Fibrillation; m, months of follow up

In this sample of 877 participants with hospital records, 595 participants had an ECG at baseline, and 609 had a detailed medication list. The participants’ characteristic are presented in Table 1. Twenty one percent of the sample (*n* = 186), 98 (26%) of Māori and 88 (18%) of non-Māori (*p* = 0.003) had AF at baseline. Period prevalence increased to 50% in Māori and 33% in non-Māori with ‘ever AF’ over 5 years (Fig. 1). Ethnic-specific incidence rates are shown in Table 2. Over 5 years, the incidence rate was 128 per 1000-person years in Māori and 54 per 1000-person years in non-Māori. Incidence increased in the first 12 months but in a downward trend from 24 months onward. At all time-points incidence of AF for Māori was double that of non-Māori, i.e. from twice the incidence rate at 12-month to 2.4 times at 5-year follow-up.

**Table 1:** Participants' characteristics at baseline

	**Whole sample, *n* = 877 (col %)**	**Māori, *n* = 379 (col %)**	**Non-Māori, *n* = 498 (col %)**
Age, mean (SD)	83.8 (2.1)	82.7 (2.8)	84.6 (0.6)
Gender,			
Men	398 (45%)	162 (42.7%)	233 (46.8%)
Women	482 (55%)	217 (57.3%)	265 (53.2%)
Education			
- Primary	189 (22%)	107 (28.9%)	82 (16.8%)
- Secondary	497 (57.9%)	211 (57.0%)	286 (58.5%)
- Trade or tertiary	173 (20.1%)	52 (14.4%)	121 (24.7%)
NZ Dep Index			
- 1 – 4 (low deprivation)	157 (17.9%)	44 (11.6%)	113 (22.7%)
- 5—7	266 (30.3%)	86 (22.7%)	180 (36.1%)
- 8—10	454 (51.8%)	249 (65.7%)	205 (41.2%)
Smoking			
- Never a smoker	414 (47.9%)	162 (43.8%)	252 (50.9%)
- Past (stopped more than 12 mths)	388 (44.9%)	169 (45.7%)	219 (44.2%)
- Current	63 (7.3%)	39 (10.5%)	24 (4.8%)
Fasting glucose (mmol/L), median (IQR)	5.3 (0.9)	5.4 (1.1)	5.2 (0.9)
FT4 (pmol/L), median (IQR)	15.2 (2.8)	15.2 (2.5)	15.1 (2.9)
TSH (mIU/L), median (IQR)	2.4 (2.0)	2.05 (1.9)	2.5 (1.9)
SBP/DBP, mean (SD)	150 (23) / 81 (13)	145 (22) / 80 (13)	152 (23) / 82 (12)
AF at baseline	186 (21.2%)	98 (25.9%)	88 (17.7%)
BP lowering medication, n (%)	460 (75.5%)	187 (82.4%)	273 (71.5%)
Statin, n (%)	229 (37.6%)	93 (41.0%)	136 (35.6%)
Aspirin	285 (32.5%)	109 (28.8%)	176 (35.3%)
Warfarin	70 (8%)	27 (7.1%)	43 (8.6%)
Digoxin	38 (6.2%)	19 (8.4%)	19 (5.0%)
Aspirin + warfarin	9 (1.5)	4 (1.8%)	5 (1.3%)
Warfarin or Aspirin	346 (56.8%)	132 (58.1%)	214 (56.0%)
Risk scores			
CHADS_2_ median (IQR)	3 (2)	3 (2)	2 (2)
CHA2DS2-Vas, median (IQR)	5 (2)	5 (2)	4 (2)
HAS-BLED, median (IQR)	3 (2)	3 (1)	3 (2)
Yearly bleeding risk, n (%)			
- 0 to 2	326 (37.2%)	169 (44.6%)	157 (31.5%)
-≥ 3 high risk	551 (62.8%)	210 (55.4%)	341 (68.5%)

*AF* Atrial fibrillation, *CHADS* Risk of stroke score, *CHADS2-Vas* modified risk of stroke score, *HAS-BLED* Risk of bleeding score, *NZ Dep Index* New Zealand Deprivation Index, *SD* Standard deviation

**Table 2:** AF incidence over 5-year follow-up in octogenarians in New Zealand

	**New cases/total person years**			**cases (95% CI) per 1000 person years**		
	**All**	**Māori**	**non-Māori**	**All**	**Māori**	**non-Māori**
**12 m**	63/707	37/297	26/409	89.1 (73.3, 108.4)	124.4 (105, 146.8)**	63.5 (50.2, 79.5)
**24 m**	153/1292	91/530	62/762	118.4 (99.5, 140.2)	171.7 (148.2, 197.6)**	81.4 (66.1, 99.6)
**36 m**	190/1781	114/712	73/1068	106.7 (88.6, 127.1)	160.0 (136.6, 186.3)**	68.3 (54.6, 85.1)
**48 m**	209/2208	126/867	83/1341	94.6 (77.8, 113.9)	145.3 (124.2, 169.5)**	61.9 (48.4, 77.2)
**60 m**	213/2579	128/997	85/1582	82.6 (67.0, 100.7)	128.4 (108.6, 151.1)**	53.7 (41.4, 68.2)

^**^ *p* < 0.001 comparing Māori and non- Māori, *AF* Atrial fibrillation, *m* = months

The 5 years period prevalence of stroke/TIA was 31% (117/379) in Māori and 34% (168/498) in non- Māori (*p* > 0.05). Period prevalence of stroke/TIA was higher in those with a history of AF than those with no AF (Fig. 1).

Overall, those with AF had more co-morbidities and were prescribed more medications than those without AF (*p* < 0.005). Cardiovascular diagnoses were more common in those with AF, as were depressive symptoms (*p* < 0.005). Those with AF had a lower functional status and physical health related quality of life (*p* < 0.05) (Table 3).

**Table 3:** Baseline characteristics of the sample of octogenarians by AF status

	**No AF, *n* = 691 (col %)**	**Yes AF, *n* = 186 (col %)**
Māori	281 (41%)	98 (53%)**
Non-Māori	410 (59%)	88 (47%)
Age, mean (SD)	84 (2)	84 (2)
Gender,		
Men	312 (45%)	83 (45%)
Women	379 (55%)	103 (55%)
Education		
- Primary	146 (21%)	43 (24%)
- Secondary	391 (58%)	106 (58%)
- Trade or tertiary	139 (21%)	34 (19%)
NZ Dep Index		
- 1 – 4 (low deprivation)	123 (18%)	34 (18%)
- 5—7	208 (30%)	58 (31%)
- 8—10	360 (52%)	94 (51%)
Smoking		
- Never a smoker	339 (50%)	75 (41%)
- Past (stopped more than 12 mths)	295 (43%)	93 (51%)
- Current	48 (7%)	15 (8%)
Alcohol		
- Never	167 (33%)	47 (39%)
- At least monthly	137 (27%)	40 (33%)
- At least twice a week	208 (41%)	33 (28%)*
Number of co-morbidities, median (IQR)*	5 (3)	6 (3)**
Conditions		
Diabetes	141 (20%)	55 (30%)*
CVD	413 (60%)	167 (90%)**
Stroke/TIA	165 (24%)	65 (35%)**
CHF	123 (18%)	107 (58%)**
Hypertension	576 (83%)	169 (91%)*
Thyroid disease	38 (5%)	11 (6%)
SBP/DBP, mean (SD)	152(23) / 81 ((12)	142 (23) / 80 (14)*
Fasting glucose (mmol/L), median (IQR)	5.30 (0.90)	5.30 (0.70)
FT4 (pmol/L), median (IQR)	15.0 (2.6)	15.8 (3.4)*
TSH (mIU/L), median (IQR)	2.5 (2.1)	3.6 (1.6)
Total med, median (IQR)*	5 (5)	7 (3)**
BP lowering medication, n (%)	354 (72%)	106 (89%)**
Statin	179 (37%)	50 (42%)
Māori with medication for AF data^#^	*N* = 177	*N* = 50
Aspirin	84 (48%)	25 (50%)
Warfarin	6 (3%)	21 (42%)
Digoxin	4 (2%)	15 (30%)
Aspirin + warfarin	nil	4 (8%)
Warfarin or Aspirin	90 (51%)	42 (84%)
Non- Māori with medication for AF data^##^	*n* = 316	*N* = 66
Aspirin	144 (46%)	32 (49%)
Warfarin	10 (3%)	33 (50%)
Digoxin	5 (2%)	14 (21%)
Aspirin + warfarin	1 (0.3%)	4 (6%)
Warfarin or Aspirin	153 (48%)	61 (92%)
Risk scores		
CHADS_2_ median (IQR)	2 (2)	3 (1)**
CHA2DS2-Vas, median (IQR)	4 (1)	6 (3)**
HAS-BLED, median (IQR)	3 (2)	3 (2)
Yearly bleeding risk, n (%)		
- 0 to 2	259 (37%)	67 (37%)
-≥ 3 high risk	435 (63%)	116 (63%)
Falls in the past 12 months	249 (36%)	79 (44%)
Function, NEALD, median (IQR)^‡^	19 (3)	18 (6)*
QoL, median (IQR) ^‡^		
- Mental health related QoL	57 (10)	56 (11)
- Physical health related QoL	44 (8)	39 (18)*
Depressive symptoms, GDS, median (IQR)^†^	2 (2)	2 (3)*
Cognition, 3MS (adjusted for visual impairment), median (IQR) ^‡^	93 (10)	92 (10)

*AF* Atrial fibrillation, *3MS* Modified mini mental state examination for cognition, *CHF* Congestive heart failure, *CHADS* Risk of stroke score, *CHADS2-Vas* modified risk of stroke score, *CVD* Cardiovascular disease, *GDS* Geriatric depression scale score for depressive symptoms, *HAS-BLED* Risk of bleeding score, *IQR* Interquartile range, *NEADL* Nottingham extended activities of daily living functional status score, *QoL* Quality of life, *SD* Standard deviation, *TIA* Transient ischaemic attack

Notes: 60 participants had no medical records of AF

^#^152 Māori had no medication details (106 in ‘no AF’ and 46 in ‘yes AF’)

^##^116 non-Māori had no medication details (95 in ‘no AF’, 21 in ‘yes AF’)

^‡^ Higher score = better function

^†^ The mean (SD) for GDS for No AF 2.2 (2.0) vs Yes AF 2.8 (2.0). Lower score = less depressive symptoms

^*^p < 0.05; ** *p* < 0.01 Adjusted for confounder (age) and effect modifier (ethnicity); interaction terms (ethnicity and exposure of interest) had p > 0.05 except for ethnicity*number of prescribed medication (*p* = 0.049); ethnicity*alcohol (*p* = 0.046) and ethnicity*DBP (*p* = 0.030)

Table 3 also shows oral anticoagulant therapy in the sample who had medication data in 2010 (*n* = 609, 227 Māori and 382 non-Māori). Forty-seven percent of those with AF (42% Māori and 50% non-Māori) took warfarin. Nearly 90% of those with AF were receiving either aspirin or warfarin (84% Māori and 92% non-Māori). Those with AF also had a higher CHADs and CHA2DS2-Vas score; HAS-BLED score was not different between those with and without AF, median (IQR) score of 3(2) (Table 3). Supplementary STable 1 (end of document) shows that over 60 months warfarin use reduced and Dabigatran use increased.

To determine the predictors of incident stroke occurrence over 5 years follow-up, Cox proportional hazard regression models were constructed excluding those with stroke at baseline (*n* = 206); regression models thus included 671 participants (546 without and 125 with AF at baseline). There were 79 primary stroke cases over 5 years (6 haemorrhagic and 73 ischaemic strokes). Atrial fibrillation was not independently associated with 5-year stroke/TIA event. There was a trend towards Māori being more likely to have a stroke/TIA event when other risks were adjusted for. Systolic blood pressure at baseline was independently associated with a higher risk of 5-year stroke/TIA event. Separate analyses including only Māori showed this SBP risk; in analyses with only non-Māori the baseline systolic blood pressure was not associated with higher risk of stroke/TIA outcome (Table 4). Separate regression analyses were completed controlling for aspirin alone, and warfarin alone (Tables 2a and 2b in supplementary materials). Results did not change significantly for either group..

**Table 4:** Multivariate Cox regression analysis for 5-year new stroke outcome in octogenarians (excluding recurrent stroke)

	**H**azard** Ratio (95%CI), *p* value**		
**Variable**	**Whole Sample**	**Māori**	**Non-Māori**
Time-varying AF	1.26 (0.62, 2.57), 0.53	1.03 (0.28, 3.77), 0.96	1.60 (0.67, 3.85), 0.29
Baseline warfarin or aspirin (ref: no)	1.35 (0.72, 2.52), 0.35	1.13 (0.43, 2.95), 0.80	1.51 (0.65, 3.48), 0.34
Age	1.11 (0.95, 1.28), 0.19	1.05 (0.89, 1.23), 0.56	1.87 (0.98, 3.56), 0.06
Gender (ref: men)	0.78 (0.45, 1.37), 0.39	0.65 (0.26, 1.62), 0.36	0.88 (0.43, 1.83), 0.74
Ethnicity (ref: non- Māori)	1.77 (0.95, 3.31), 0.07	-	-
NZDep med (ref = high)	0.67 (0.33, 1.35), 0.26	0.65 (0.20, 2.15), 0.49	0.61 (0.25, 1.49), 0.28
NZDep low (ref = high)	0.54 (0.27, 1.09), 0.09	0.39 (0.13, 1.21), 0.10	0.67 (0.28, 1.62), 0.37
Baseline SBP	1.01 (1.00, 1.03), 0.03	1.02 (1.00, 1.04), 0.03	1.01 (0.99, 1.02), 0.25
Statin (ref = no)	0.73 (0.39, 1.39), 0.34	1.03 (0.38, 2.79), 0.95	0.58 (0.24, 1.35), 0.20
CHF, prior (ref = no)	0.75 (0.32, 1.73), 0.45	0.35 (0.08, 1.61), 0.18	1.21 (0.43, 3.36), 0.72

*NZDep* New Zealand deprivation index

*Ref* Reference

*SBP* Systolic blood pressure

*CHF* Congestive heart failure

Over the course of 5 years follow-up 189 Māori had died from any cause compared with 184 non-Māori (45% and 36% respectively: *p* = 0.004). Table 5 shows AF was independently associated with 5-year mortality risk, and in separate analyses by ethnicity, the risk was observed in Māori only [HR 2.29 (95% CI 1.37–3.82, *p* < 0.01)]. Risk factors associated with mortality differed between Māori and non- Māori. In addition to AF, older age, male gender and having CHF at baseline increased mortality and receiving statins was associated with reduced mortality (Table 4). For non-Māori, AF was not independently associated with mortality [HR 1.12 (95% CI 0.67–1.87, *p* = 0.66)]; male gender, living in an area of low deprivation, and having CHF at baseline were associated with increased mortality. The use of warfarin or aspirin at baseline had an apparent opposite impact in Māori (protective) and non- Māori (not protective), non-significantly. Separate regression analyses were completed controlling for aspirin alone, and warfarin alone (STables 3a and b in supplementary materials). The association between AF and 5-year mortality in these models did not change significantly; warfarin alone was associated with a higher mortality risk in non-Māori.

**Table 5:** Multivariate Cox regression analysis for 5-year mortality of octogenarians

**H**azard** R**atio** (95% C**onfidence** Interval), *p* value**			
**Variable**	**Whole Sample**	**Māori**	**Non-Māori**
Time-varying AF	1.56 (1.10, 2.22), 0.01	2.29 (1.37, 3.82), < 0.01	1.12 (0.67, 1.87), 0.66
Baseline warfarin or aspirin (ref: no)	1.09 (0.76, 1.55), 0.65	0.64 (0.38, 1.09), 0.10	1.60 (0.98, 2.60), 0.06
Age	1.17, (1.08, 1.26), < 0.01	1.16 (1.06, 1.26), < 0.01	1.34 (0.93, 1.94), 0.12
Gender (ref: men)	0.55 (0.41, 0.75), < 0.01	0.56 (0.34, 0.91), 0.02	0.54 (0.36, 0.81), < 0.01
Ethnicity (ref: non-Māori)	1.45 (1.03, 2.03), 0.03	-	-
NZDep med (ref = high)	1.06 (0.66, 1.71), 0.80	0.79 (0.34, 1.83), 0.59	1.16 (0.65, 2.06), 0.61
NZDep low (ref = high)	1.77 (1.15, 2.74), 0.01	1.18 (0.57, 2.43), 0.66	2.06 (1.20, 3.53), < 0.01
Baseline SBP	1.00 (0.99, 1.01), 0.72	1.00 (0.99, 1.01), 0.99	1.00 (0.99, 1.01), 0.69
Statin (ref = no)	0.66 (0.47, 0.93),0.02	0.49 (0.29, 0.84), < 0.01	0.76 (0.49, 1.19), 0.24
CHF, prior (ref = no)	2.21 (1.53, 3.18), < 0.01	2.56 (1.47, 4.44), < 0.01	1.96 (1.19, 3.23), 0.01

*AF Atrial fibrillation*

*NZDep* New Zealand deprivation index

*Ref* Reference

*SBP* Systolic blood pressure

*CHF* Congestive heart failure

## Discussion

This study reports the prevalence and incidence rate of AF and risk of stroke and mortality in a population-based sample of octogenarians roughly representing the underlying population on demographics and smoking status [28, 29]. In this longitudinal study, with 5 years of follow up, we found that the prevalence of AF continued to increase with age. More Māori than non-Māori (mostly European descent) had AF by their 5-year follow-up (over half and up to a third respectively). Our findings extend the current NZ [3, 14] and international literature [1, 2] and is the first study to report the high prevalence of AF in Māori aged 80 + years.

The AF incidence rate observed in this cohort study was higher than previously reported. In the Rotterdam Study of 960 adults aged 80 + , the incidence rate was 20.7/1000 and 18.2/1000 person-years for those aged 80–84 and ≥ 85 years respectively [1]. The ascertainment methods seemed similar to the current report, but the quality of medical records could be influenced by the physicians and health system emphasis on recording AF. In Canada incidence rate was 31.7/1000 person years for those aged 80 + , with a higher prevalence in women (41.5%) than men (24.3%) [10]. In a large German study of administrative data the incidence was 31/1000 person years with no difference between men and women [2]. In contrast to the Cardiovascular Health Study, where the incidence rate was higher in men than women aged ≥ 80 years, 58.7 and 25.1 per 1000 person-years respectively [1], we did not observe a difference between men and women in the current study but our rates are high at 128 and 53/1000 person years for Māori and non-Māori respectively.

Importantly, in the current study identified inequity in incidence as Māori had twice that of non-Māori, which may be explained at least in part by different co-morbidity rates between the ethnic groups. This includes, for example, higher prevalence of CHF and diabetes among Māori compared with non-Māori [29]. Overall, the very high prevalence of AF among older Māori and the relationship with AF and mortality supports a need for greater focus on screening and AF management for older Māori as well as broader investigations that can further explain the difference in incidence.

The lower proportion of Māori receiving anti-coagulation suggests under-treatment of AF among Māori. Disproportionate access to cardiovascular disease treatments in Māori [36] is one of the many equity issues contributing to disparity in mortality for Māori. Further disparities in heart failure and ischemic heart disease are described [36, 37] and the current study adds ethnic bias in treatment of AF in old age. We also acknowledge under-treatment may be confounded by indication. Overall, the management for AF in older people is challenging. In this sample, all participants had at least 4% annual stroke risk and this risk increased up to 10% for those with AF suggesting treatment is needed for all octogenarians with AF. However, older people also have an increased risk of bleeding. With the availability of new oral anticoagulant such as dabigatran (available in NZ from 2012) with lower bleeding rates but similar efficacy as warfarin [38], risks of AF treatment with anticoagulation may be more acceptable to general practitioners, who are the main prescribers for older people.

We did not find an independent association between AF and 5-year new stroke/TIA risk. It is possible that the impact of AF on stroke risk in octogenarians is a co-occurring with other morbidities. Patterns of co-morbidities in this cohort were more closely related to hospitalisations and death than number of conditions [39], and while those with the combination of AF and CHF had a higher risk of 48-month hospitalisation [39], the cause of hospitalisation was not examined. We did not find that AF was associated with cognitive decline; [40, 41] though we concur that AF is associated with reduced functional capacity and quality of life [42]. We acknowledge the null association observed with stroke/TIA outcome could be a Type II error and recommend further studies to examine the cause and impact of AF in octogenarians; it is possible that the AF and stroke association is not similar in advanced age as observed in the younger population.

The ARCOS studies of stroke in NZ show Māori have stroke at younger age [43], and have differing patterns of subtypes and risk factor profiles [44]. However the disparity in stroke incidence was not observed after age 85 years. This is consistent with our current data. In our whole group analyses, increasing blood pressure was an independent risk for stroke/TIA, mostly driven by the specific analyses for Māori. These findings are consistent with national [44] and international [45] data of the importance of increased blood pressure with risk of stroke/TIA. These findings have direct relevance for Māori and reiterate the need for strategies to improve blood pressure management for Māori.

The current study confirms a clear ethnic inequity in all-cause mortality: AF was strongly associated with mortality for Māori, independent of the presence of CHF, whereas for non-Māori, CHF was associated with mortality, but AF was not once anti-coagulants and other treatments were taken into account. Interestingly the risk associated with anticoagulant treatment was high for non- Māori (indication bias) but low for Māori. While both associations are non-significant, the much reduced use of warfarin in this sample is worthy of further examination. AF and heart failure both occur in the context of similar cardiovascular risk factors and clinical CVD. At baseline, almost half of those with AF had co-existing heart failure, and AF has been associated with worse outcome for patients with established CHF. Perhaps this sample of advanced age Māori showed a glimpse of the impact of earlier exposure to AF among Māori at a younger age [14]. Our data reinforce the importance of both AF and heart failure and highlight opportunities for improving outcomes by addressing these specific conditions. Statin treatment is potentially a proxy for adherence to guidelines for management of CVD [46], and in this analyses appeared protective for Māori.

Socioeconomic deprivation was associated with mortality risk for non-Māori but not Māori. Māori in the LiLACS NZ cohorts live in more deprived areas [26]. Potentially, disparities in cardiovascular disease [36, 37] and its management in addition to the unquantifiable impacts of colonisation are stronger drivers of mortality than deprivation alone. The current study, with differing risk factors associated with both stroke and mortality for Māori than non- Māori in advanced age, supports ethnic specific studies and strategies to untangle the health inequalities in NZ [47]. For non-Māori the finding that higher mortality risk was associated with residence in an area of low deprivation challenges the inverse care law. It may be that those managing to live to an old age in high deprivation areas are in some way more resilient.

### Strength and limitations

This strength of this study is the population based sampling frame, equal sampling and engagement with indigenous elders with a similar response rates to non-indigenous. The response rate of < 60% and the number with full data available was lower than ideal, however we have complete ascertainment of stroke, and multiple sources of information about AF in this sample. The detail of the data collected and accuracy of ascertainment of outcomes means these associations identified are very likely robust. While this study provides some evidence on the burden of AF in these groups engaged in a cohort study, it also presents several limitations including the relatively small number of events and overall sample size. The ethnic-specific Cox models in this study may have reduced statistical power for differentiating the independent influences of AF and heart failure on the outcomes. We recommend cautious interpretation of the results.

## Conclusion

In conclusion, AF is highly prevalent in octogenarians, particularly in indigenous people and continues to increase with age. Under-treatment of AF in octogenarians is also prevalent and may be attributed to the challenges presented to physicians. The impact of AF on long-term health outcomes is likely to be multifaceted and further research is needed to examine this in more detail to facilitate clinical decision weighing up the risk and benefit of treating AF in octogenarians.

## Supplementary Information

**Additional file 1:** **STable 1.** number of participants prescribed with anticoagulant over 5 years follow-up. **STable 2a.** Multivariate Cox regression analysis for 5-year new stroke outcome in octogenarians (excluding recurrent stroke). **STable 2b.** Multivariate Cox regression analysis for 5-year new stroke outcome in octogenarians (excluding recurrent stroke). **STable 3a.** Multivariate Cox regression analysis for 5-year mortality of octogenarians. **STable 3b.** Multivariate Cox regression analysis for 5-year mortality of octogenarians.

## Data Availability

The datasets used and/or analysed during the current study are available from the corresponding author after appropriate permission from the Māori oversight committee is obtained.
