# Supplementary material for: Atrial fibrillation incidence and outcomes in two cohorts of octogenarians: LiLACS NZ
Source: BMC Geriatr. 2023 Mar 30;23:197. doi: 10.1186/s12877-023-03902-5 (PMC10064671; doi:10.1186/s12877-023-03902-5)
Supplement: Supplementary file 1 — Additional file 1: STable 1. number of participants prescribed with anticoagulant over 5 years follow-up. STable 2a. Multivariate Cox regression analysis for 5-year new stroke outcome in octogenarians (excluding recurrent stroke). STable 2b. Multivariate Cox regression analysis for 5-year new stroke outcome in octogenarians (excluding recurrent stroke). STable 3a. Multivariate Cox regression analysis for 5-year mortality of octogenarians. STable 3b. Multivariate Cox regression analysis for 5-year mortality of octogenarians. [file 12877_2023_3902_MOESM1_ESM.docx]

## Atrial fibrillation incidence and outcomes in two cohorts of octogenarians: LiLACS NZ - Supplementary material

Ruth Teh^1^, Ngaire Kerse^1^, Avinesh Pillai^2^, Thomas Lumley^2^, Anna Rolleston^3^, Tin Aung Kyaw^1^, Martin Connolly,^4^ Joanna Board,^4^ Elaine Monteiro^1^, Valerie Wright-St Clair,^5^ Robert N Doughty^6^

1. Department of General Practice and Primary Health Care, Faculty of Medical and Health Sciences, University of Auckland, Auckland, New Zealand
2. Department of Statistics, Faculty of Science, University of Auckland, Auckland, New Zealand
3. Manawa Ora, The Centre for Health, Tauranga, New Zealand
4. Department of Geriatric Medicine, Faculty of Medical and Health Sciences, University of Auckland, Auckland, New Zealand
5. Centre for Active Ageing, Auckland University of Technology New Zealand
6. Department of Medicine, University of Auckland and Greenlane Cardiovascular Service, Auckland District Health Board

*Corresponding author*

Ruth Teh, Department of General Practice and Primary Health Care, Faculty of Medical and Health Sciences, University of Auckland, PO Box 92019, Auckland, New Zealand

[r.teh@auckland.ac.nz](mailto:r.teh@auckland.ac.nz)

## Supplementary material

STable 1: number of participants prescribed with anticoagulant over 5 years follow-up

|  | **Warfarin** | **Acetylsalicylic acid** | **Dabigatran etexilate** | **Rivaroxaban** | **digoxin** |
| --- | --- | --- | --- | --- | --- |
| Baseline | 71 | 296 |  |  | 39 |
| 12 months | 59 | 283 | 9 |  | 48 |
| 24 months | 41 | 202 | 15 |  | 30 |
| 36 months | 35 | 147 | 20 |  | 27 |
| 48 months | 29 | 108 | 18 |  | 18 |
| 60 months | 23 | 76 | 17 | 2 | 19 |

STable 2a: Multivariate Cox regression analysis for 5-year new stroke outcome in octogenarians (excluding recurrent stroke)

|  | **Hazard Ratio (95%CI), p value** | | |
| --- | --- | --- | --- |
| **Variable** | **Whole Sample** | **Māori** | **Non-Māori** |
| Ethnicity (ref: non- Māori) | 1.76 (0.94, 3.28), 0.08 |  |  |
| Time-varying AF | 1.37 (0.67, 2.78), 0.38 | 1.07 (0.29,3.81), 0.92 | 1.88 (0.79, 4.52), 0.16 |
| Baseline aspirin alone (ref: no) | 1.218 (0.67, 2.21), 0.52 | 0.94 (0.36,2.42), 0.89 | 1.53 (0.70, 3.34), 0.29 |
| Age | 1.11 (0.95, 1.28), 0.186 | 1.05 (0.89,1.23), 0.55 | 1.90 (1.00, 3.60), 0.05 |
| Gender (ref: men) | 0.78 (0.45, 1.36), 0.38 | 0.65 (0.26,1.61), 0.35 | 0.89 (0.43, 1.83), 0.74 |
| NZDep med (ref=high) | 0.68 (0.34, 1.38), 0.29 | 0.40 (0.13,1.2), 0.104 | 0.68 (0.28, 1.64), 0.391 |
| NZDep low (ref=high) | 0.55 (0.27, 1.11), 0.10 | 1.02 (1.00,1.04), 0.03 | 1.01 (0.99, 1.02), 0.265 |
| Baseline SBP | 1.01 (1.00, 1.02), 0.03 | 1.12 (0.41,3.03), 0.83 | 0.57 (0.24, 1.34), 0.20 |
| Statin (ref=no) | 0.77 (0.41, 1.44), 0.41 | 0.36 (0.08,1.64), 0.19 | 1.28 (0.47, 3.48), 0.63 |
| CHF, prior (ref=no) | 0.79 (0.34, 1.81), 0.58 | 1.076 (0.30,3.8), 0.92 | 1.88 (0.79, 4.51), 0.16 |

STable 2b: Multivariate Cox regression analysis for 5-year new stroke outcome in octogenarians (excluding recurrent stroke)

|  | **Hazard Ratio (95%CI), p value** | | |
| --- | --- | --- | --- |
| **Variable** | **Whole Sample** | **Māori** | **Non-Māori** |
| Ethnicity (ref: non- Māori) | 1.73 (0.92, 3.23), 0.09 |  |  |
| Time-varying AF | 1.42 (0.69, 2.93), 0.34 | 0.94 (0.26, 3.49), 0.9304 | 2.22 (0.92, 5.34), 0.08 |
| Baseline warfarin alone (ref: no) | 0.66 (0.20, 2.21), 0.50 | 2.75 (0.57, 13.25), 0.207 | 0.22 (0.03, 1.72), 0.15 |
| Age | 1.10 (0.95, 1.28), 0.20 | 1.06 (0.90, 1.24), 0.50 | 1.90 (1.0, 3.59), 0.05 |
| Gender (ref: men) | 0.78 (0.45, 1.36), 0.38 | 0.66 (0.26, 1.64), 0.37 | 0.87 (0.41, 1.80), 0.71 |
| NZDep med (ref=high) | 0.71 (0.35, 1.42), 0.33 | 0.38 (0.12, 1.17), 0.09 | 0.70 (0.29, 1.71), 0.44 |
| NZDep low (ref=high) | 0.56 (0.28, 1.12), 0.10 | 1.02 (1.00, 1.04), 0.02 | 1.01 (0.99, 1.02), 0.31 |
| Baseline SBP | 1.01 (1.00, 1.02), 0.03 | 1.04 (0.42, 2.60), 0.93 | 0.71 (0.32, 1.57), 0.40 |
| Statin (ref=no) | 0.84 (0.46, 1.51), 0.55 | 0.32 (0.07, 1.51), 0.15 | 1.45 (0.53, 3.94), 0.47 |
| CHF, prior (ref=no) | 0.83 (0.36, 1.90), 0.65 | 0.94 (0.26, 3.49), 0.93 | 2.22 (0.92, 5.34), 0.08 |

STable 3a: Multivariate Cox regression analysis for 5-year mortality of octogenarians

|  | **Hazard Ratio (95%CI), p value** | | |
| --- | --- | --- | --- |
| **Variable** | **Whole Sample** | **Māori** | **Non-Māori** |
| Ethnicity (ref: non- Māori) | 1.45 (1.04, 2.03), 0.03 |  |  |
| Time-varying AF | 1.64 (1.16, 2.33), 0.01 | 2.12 (1.28, 3.50), <0.01 | 1.31 (0.78, 2.19), 0.31 |
| Baseline aspirin alone (ref: no) | 1.24 (0.91, 1.70), 0.18 | 1.05 (0.65, 1.69), 0.85 | 1.37 (0.88, 2.13), 0.16 |
| Age | 1.17 (1.08, 1.26), <0.01 | 1.15 (1.06, 1.25), <0.01 | 1.35 (0.93, 1.95), 0.11 |
| Gender (ref: men) | 0.55 (0.41, 0.758), <0.01 | 0.60 (0.37, 0.97), 0.04 | 0.55 (0.36, 0.82), <0.01 |
| NZDep med (ref=high) | 1.059 (0.66, 1.70), 0.81 | 1.12 (0.54, 2.33), 0.76 | 2.06 (1.20, 3.53), <0.01 |
| NZDep low (ref=high) | 1.75 (1.13, 2.71), <0.01 | 1.00 (0.99, 1.01), 0.85 | 1.0 (0.99, 1.017), 0.60 |
| Baseline SBP | 1.00 (0.99, 1.01), 0.66 | 0.45 (0.26, 0.76), <0.01 | 0.80 (0.51, 1.25), 0.33 |
| Statin (ref=no) | 0.64 (0.46, 0.89), 0.01 | 2.25 (1.34, 3.79), <0.01 | 2.13 (1.31, 3.47), <0.01 |
| CHF, prior (ref=no) | 2.21 (1.56, 3.13), <.01 | 2.12 (1.28, 3.50), <0.01 | 1.31 (0.78, 2.19), 0.31 |

STable 3b: Multivariate Cox regression analysis for 5-year mortality of octogenarians

|  | **Hazard Ratio (95%CI), p value** | | |
| --- | --- | --- | --- |
| **Variable** | **Whole Sample** | **Māori** | **Non-Māori** |
| Ethnicity (ref: non- Māori) | 1.46 (1.04, 2.04), 0.03 |  |  |
| Time-varying AF | 1.54 (1.08, 2.20), 0.02 | 2.18 (1.32, 3.61), <0.01 | 0.96 (0.55, 1.68), 0.89 |
| Baseline warfarin alone (ref: no) | 1.19 (0.72, 1.95), 0.50 | 0.50 (0.19, 1.33), 0.17 | 2.18 (1.17, 4.06), 0.01 |
| Age | 1.17 (1.08, 1.27), <0.01 | 1.15 (1.05, 1.25), <0.01 | 1.23 (0.85, 1.80), 0.27 |
| Gender (ref: men) | 0.56 (0.41, 0.76), <0.01 | 0.58 (0.36, 0.94), 0.03 | 0.56 (0.37, 0.84), <0.01 |
| NZDep med (ref=high) | 1.07 (0.67, 1.71), 0.78 | 0.73 (0.31, 1.68), 0.45 | 1.17 (0.66, 2.08), 0.59 |
| NZDep low (ref=high) | 1.79 (1.16, 2.76), <0.01 | 1.078(0.52, 2.23), 0.84 | 2.11 (1.23, 3.60), <0.01 |
| Baseline SBP | 1.0 (0.99, 1.01), 0.75 | 1.00 (0.99, 1.01), 0.87 | 1.00 (0.99, 1.01), 0.599 |
| Statin (ref=no) | 0.67 (0.49, 0.93), 0.02 | 0.47 (0.28, 0.79), <0.01 | 0.84 (0.55, 1.28), 0.41 |
| CHF, prior (ref=no) | 2.25 (1.59, 3.20), <.01 | 2.41 (1.43, 4.07), <0.01 | 2.31 (1.42, 3.78), <0.01 |
